# Infective Endocarditis in Pregnancy Diagnosed Early Based on Elevated D-Dimer Levels: A Case Report

**DOI:** 10.7759/cureus.61803

**Published:** 2024-06-06

**Authors:** Kazuki Yamano, Akira Kitano, Fumika Hamaguchi, Michikazu Nagura, Masataka Nakajima

**Affiliations:** 1 Obstetrics and Gynecology, Nagahama Red Cross Hospital, Nagahama, JPN

**Keywords:** pregnancy and heart disease, infective endocarditis, elevated d-dimer, clinical chorioamnionitis, positive blood culture, staphylococcus aureus bacteremia

## Abstract

We discuss a case where the blood cultures of a patient with clinical chorioamnionitis and elevated D-dimer levels enabled early diagnosis of infective endocarditis. A 31-year-old female with a 39-week pregnancy presented to the obstetrics department with a fever. Cardiotocography revealed fetal tachycardia and severe late deceleration. Preoperative examinations revealed a leukocyte count of 15,900/μL and D-dimer levels of 86.2 μg/mL. She was diagnosed with a non-reassuring fetal status due to clinical chorioamnionitis; accordingly, an emergency cesarean section was performed. Imaging studies ruled out the possibility of a thromboembolism. Subsequent maternal blood cultures were positive for *Staphylococcus aureus*. Echocardiography revealed vegetation on the aortic valve, leading to a diagnosis of infective endocarditis. Blood cultures can be useful in evaluating for sepsis in cases of clinical chorioamnionitis with elevated D-dimer levels as they may facilitate early diagnosis of infective endocarditis during pregnancy.

## Introduction

Infective endocarditis during pregnancy is a rare entity and may present with the clinical features of clinical chorioamnionitis. Infective endocarditis is often diagnosed in the advanced stages, and established methods for early diagnosis are lacking. Blood cultures, which are important for the diagnosis of infective endocarditis, are generally not performed for clinical chorioamnionitis. We report a case in which blood cultures for clinical chorioamnionitis with elevated D-dimer levels enabled the early diagnosis of infective endocarditis.

## Case presentation

The patient was a 31-year-old woman (gravida 1, para 0) who had an established spontaneous pregnancy that was confirmed by her previous obstetrician. Accordingly, she continued her prenatal checkups. Her medical history included atopic dermatitis; however, her family history was unremarkable. She had no history of invasive treatments, including dental treatments, during pregnancy. Membrane sweeping was performed at 39 weeks and one day of gestation. Irregular painful uterine contractions persisted for several days. She visited her previous obstetrician with a chief complaint of a fever of 39 °C at 39 weeks and six days of gestation. She had no subjective symptoms other than fever.

**Figure 1:**
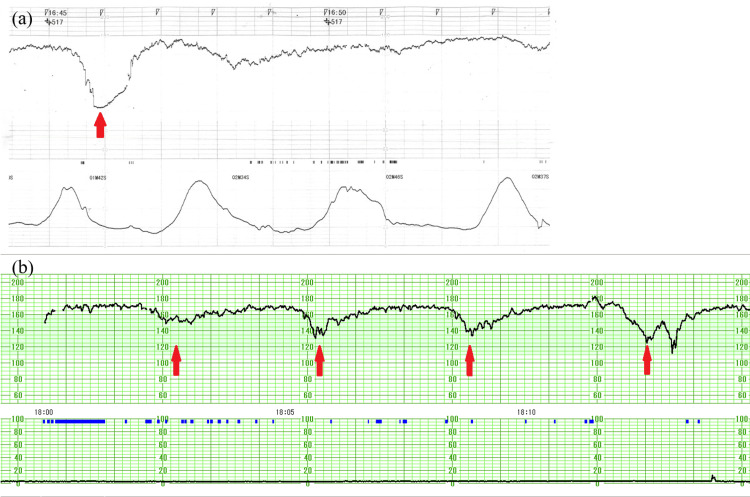
Cardiotocography showing fetal tachycardia and severe late deceleration (red arrow) (category II) in both the previous obstetric record (a) and our hospital record (b)

Cardiotocography revealed uterine contractions every few minutes, fetal tachycardia, and severe late deceleration (category II) (Figure 1a). She was transported to our hospital with a non-reassuring fetal status. During transport, her vital signs showed a temperature of 37.5 °C, pulse of 103/min, blood pressure of 88/52 mmHg, respiratory rate of 23/min, and SpO_2_ of 99% (oxygen 10 L/min). The quick sequential organ failure assessment (SOFA) score was 2 points. She underwent preoperative examinations such as blood tests, chest radiography, and electrocardiography. Blood tests revealed a white blood cell count of 15,900/μL (reference range: 3000-8500/μL), a platelet count of 147,000/μL (reference range: 150,000-350,000/μL), C-reactive protein level of 0.52 mg/dL (reference range: 0.0-0.5 mg/dL), and D-dimer level of 86.2 μg/mL (reference range: <1.0 μg/mL). No other remarkable preoperative findings were observed. Rapid tests for group A streptococcus, influenza, and coronavirus disease 2019 (COVID-19) were all negative. She met the Lencki criteria [maternal fever (>38 ℃), maternal tachycardia (>100/min), and elevated white blood cell count (>15,000/μL)] and was diagnosed with clinical chorioamnionitis. Also, based on these findings and cardiotocography findings at our hospital (Figure 1b), she was diagnosed with a non-reassuring fetal status due to clinical chorioamnionitis.

**Figure 2:**
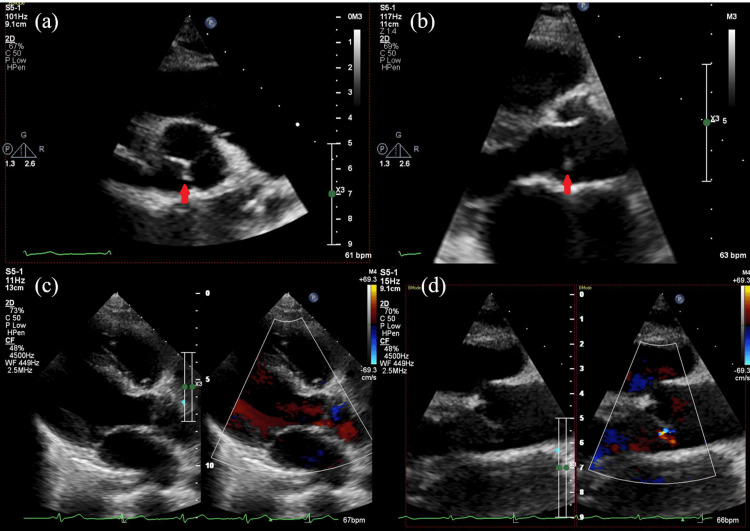
Transthoracic echocardiography at the beginning of treatment revealing vegetation on the aortic valve (red arrow) (a) (b) and mild aortic regurgitation (c) (d)

**Figure 3:**
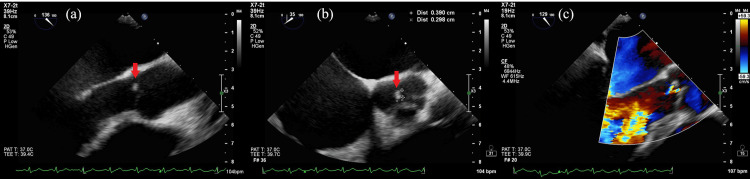
Transesophageal echocardiography at the beginning of treatment revealing vegetation on the aortic valve (red arrow) (a) (b) and mild aortic regurgitation (c)

Cervical ripening was poor, and her water was yet to break. Early vaginal delivery was not expected; accordingly, an emergency cesarean section was performed. Intraoperatively, the amniotic fluid was turbid. An intraoperative culture test of amniotic fluid swabs and a blood culture of the newborn were performed. Given the elevated D-dimer levels, contrast-enhanced CT was performed immediately after the cesarean section, and thromboembolism was ruled out. Sepsis was the suspected cause of the elevated D-dimer levels, and two sets of maternal blood cultures were submitted. The patient was treated with ampicillin and azithromycin for clinical chorioamnionitis. The two sets of maternal blood cultures, amniotic fluid swab culture, and neonatal blood culture all detected *Staphylococcus aureus* with good sensitivity. Accordingly, the antibiotic regimen was changed to cefazolin; moreover, maternal echocardiography was planned since the detected microorganisms are common causative agents of infective endocarditis. Transthoracic echocardiography revealed vegetation on the aortic valve and mild aortic regurgitation (Figure 2), with transesophageal echocardiography yielding similar findings (Figure 3).

**Figure 4:**
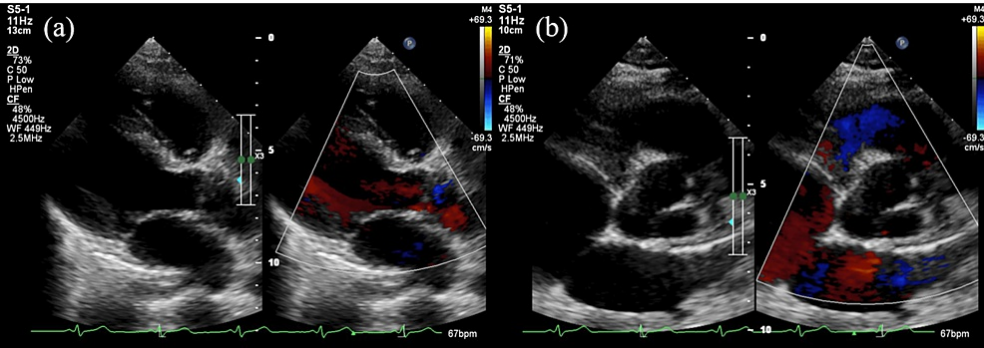
Transthoracic echocardiography at the end of treatment showing that the vegetation on the aortic valve is resolved but trivial aortic regurgitation remains (a) (b)

She met two major modified Duke criteria [major microbiology criteria (positive blood cultures) and major imaging criteria (echocardiography)] and was diagnosed with infective endocarditis. She underwent an oral examination by a dentist; however, no obvious caries or periodontal disease was noted. Three days after antibiotic initiation, blood culture tests were repeated, and they yielded negative results. Subsequently, the antibiotic therapy was continued for six weeks. The D-dimer levels were 25.7 μg/mL, 10.0 μg/mL, and 1.1 μg/mL at two, six, and 20 days after antibiotic initiation, respectively, confirming a decreasing trend. The patient completed treatment and was discharged with only trivial aortic regurgitation (Figure 4) and no fever flare-up. Since discharge, the patient has not experienced flare-ups of infective endocarditis or heart failure.

She delivered a female baby with no anomalies, who weighed 3082 g, and had Apgar scores of 8 and 9 at one and five minutes, respectively. Umbilical artery pH was 7.300 and base excess was -4.3 mmol/L. She was admitted to the neonatal ICU (NICU) with a diagnosis of bacteremia caused by *Staphylococcus aureus* based on blood culture and had no vegetation on echocardiography. She was started on antibiotic therapy for 10 days and was discharged from the NICU at the age of 14 days.

The placenta and umbilical cord were submitted for pathological examination, which revealed chorioamnionitis stage 3 and umbilical cord inflammation stage 3.

## Discussion

This case report highlights two key points. Firstly, blood culture tests should be considered to evaluate for sepsis in patients with clinical chorioamnionitis who have elevated D-dimer levels. Secondly, the detection of elevated D-dimer levels may aid in the early detection of infective endocarditis during pregnancy. In general, blood cultures are not performed for patients with clinical chorioamnionitis. However, sepsis can be suspected in cases of systolic blood pressure <90 mmHg or >160 mmHg, diastolic blood pressure >100 mmHg, heart rate <50/min or >120/min, or respiratory rate <10/min or >30/min during pregnancy [1]. Additionally, the quick SOFA score seems a useful parameter for the early detection of sepsis in adults [2].

In our case, the quick SOFA score was 2 points, indicating possible sepsis. Moreover, blood cultures were performed, given the markedly elevated D-dimer levels on preoperative examinations. The reference ranges for D-dimer levels are 0.05-0.95 μg/mL or 0.2-0.9 μg/mL in the first trimester of pregnancy, 0.32-1.29 μg/mL or 0.2-1.5 μg/mL in the second trimester, and 0.13-1.7 μg/mL or 0.4-2.8 μg/mL in the third trimester [3,4]. The levels are often higher than those in non-pregnant women. Additionally, sepsis has been reported to occur in 24% of non-pregnant women with D-dimer levels >5 μg/mL [5]. Accordingly, if patients with clinical chorioamnionitis present with D-dimer levels >5 μg/mL on preoperative examination, blood cultures should be considered to assess for sepsis. In our case, the SOFA score was at least 3 points in total, with 1 point for coagulation, 1 point for cardiovascular, and at least 1 point for respiration based on oxygen administration and SpO_2_, although the lack of PaO_2_ measurements made accurate assessment difficult. Accordingly, a diagnosis of sepsis was made.

While infective endocarditis during pregnancy is very rare (0.006%), it is a potentially serious disease with mortality rates of 33% and 29% for mothers and infants, respectively [6,7]. Therefore, early detection is important. D-dimer levels increase during the early stage of bacteremia, and D-dimer levels exceeding 4 μg/mL increase the risk of in-hospital mortality [8]. In infective endocarditis, D-dimer levels increase to 3.2 μg/mL, with the risk of in-hospital mortality increasing above 4.2 μg/mL [9]. In our case, the combination of pregnancy, sepsis, and infective endocarditis may have caused the markedly elevated D-dimer levels. A decrease in D-dimer levels was observed throughout the treatment course.

Since elevated D-dimer levels occur in the early stages of bacteremia before infective endocarditis, higher elevated D-dimer levels may be observed in the early stages of infective endocarditis during pregnancy. Accordingly, blood cultures should be performed in cases of fever accompanied by high D-dimer levels. Moreover, immediate echocardiography is recommended if the microorganisms that commonly cause infective endocarditis are detected, which include *Staphylococcus aureus*, *Staphylococcus lugdunensis*, *Enterococcus faecalis*, all streptococcal species (except *Streptococcus pneumoniae* and *Streptococcus pyogenes*), *Granulicatella* spp., *Abiotrophia* spp., *Gemella* spp., and HACEK group microorganisms [10]. In *Staphylococcus aureus* bacteremia, 13% of cases are complicated by infective endocarditis [11]. Therefore, routine echocardiography is recommended even in the absence of chest symptoms.

In the present case, the cause of infective endocarditis was unclear. Atopic dermatitis may be an underlying disease of infective endocarditis caused by *Staphylococcus aureus* [12], which may be pertinent in our case. However, we did not observe skin lesions that could have been an entry point for bacteremia; moreover, the patient had no history of invasive treatment, including dental treatment. Amniotomy does not increase the risk of intrauterine infection [13], and the causal relationship between membrane sweeping and infection in our case remains unclear.

In cases of clinical chorioamnionitis with elevated D-dimer levels, blood cultures may be considered to evaluate for sepsis, which may facilitate the early detection of infective endocarditis during pregnancy. In our case, the mother and baby survived without major complications. However, if the antibiotic treatment regimen had been stopped at the acute stage of clinical chorioamnionitis, the mother may have been diagnosed with advanced infective endocarditis at a later date. Thus, when handling cases of elevated D-dimer levels on preoperative examination in patients with clinical chorioamnionitis, it is important to not only consider thromboembolism but also take sepsis and infective endocarditis into account.

## Conclusions

Infective endocarditis during pregnancy may present with the clinical features of clinical chorioamnionitis. Blood cultures, which are important for infective endocarditis diagnosis, are generally not performed for clinical chorioamnionitis. In cases of clinical chorioamnionitis with elevated D-dimer levels, blood cultures may be considered to evaluate for sepsis, which may facilitate the early detection of infective endocarditis during pregnancy. When handling cases of elevated D-dimer levels on preoperative examination in patients with clinical chorioamnionitis, it is important to include thromboembolism, sepsis, and infective endocarditis in the differential diagnosis.
